# PSORTdb 4.0: expanded and redesigned bacterial and archaeal protein subcellular localization database incorporating new secondary localizations

**DOI:** 10.1093/nar/gkaa1095

**Published:** 2020-12-11

**Authors:** Wing Yin Venus Lau, Gemma R Hoad, Vivian Jin, Geoffrey L Winsor, Ashmeet Madyan, Kristen L Gray, Matthew R Laird, Raymond Lo, Fiona S L Brinkman

**Affiliations:** Department of Molecular Biology and Biochemistry, Simon Fraser University, Burnaby, British Columbia V5A 1S6, Canada; Department of Molecular Biology and Biochemistry, Simon Fraser University, Burnaby, British Columbia V5A 1S6, Canada; Department of Molecular Biology and Biochemistry, Simon Fraser University, Burnaby, British Columbia V5A 1S6, Canada; Department of Molecular Biology and Biochemistry, Simon Fraser University, Burnaby, British Columbia V5A 1S6, Canada; Department of Molecular Biology and Biochemistry, Simon Fraser University, Burnaby, British Columbia V5A 1S6, Canada; Department of Molecular Biology and Biochemistry, Simon Fraser University, Burnaby, British Columbia V5A 1S6, Canada; Department of Molecular Biology and Biochemistry, Simon Fraser University, Burnaby, British Columbia V5A 1S6, Canada; Department of Molecular Biology and Biochemistry, Simon Fraser University, Burnaby, British Columbia V5A 1S6, Canada; Department of Molecular Biology and Biochemistry, Simon Fraser University, Burnaby, British Columbia V5A 1S6, Canada

## Abstract

Protein subcellular localization (SCL) is important for understanding protein function, genome annotation, and aids identification of potential cell surface diagnostic markers, drug targets, or vaccine components. PSORTdb comprises ePSORTdb, a manually curated database of experimentally verified protein SCLs, and cPSORTdb, a pre-computed database of PSORTb-predicted SCLs for NCBI’s RefSeq deduced bacterial and archaeal proteomes. We now report PSORTdb 4.0 (http://db.psort.org/). It features a website refresh, in particular a more user-friendly database search. It also addresses the need to uniquely identify proteins from NCBI genomes now that GI numbers have been retired. It further expands both ePSORTdb and cPSORTdb, including additional data about novel secondary localizations, such as proteins found in bacterial outer membrane vesicles. Protein predictions in cPSORTdb have increased along with the number of available microbial genomes, from approximately 13 million when PSORTdb 3.0 was released, to over 66 million currently. Now, analyses of both complete and draft genomes are included. This expanded database will be of wide use to researchers developing SCL predictors or studying diverse microbes, including medically, agriculturally and industrially important species that have both classic or atypical cell envelope structures or vesicles.

## INTRODUCTION

Protein subcellular localization (SCL) prediction aids characterization of a protein's biological function and assists in gene annotation of microbial sequences. Bacterial and archaeal proteins can be free-floating in the cytoplasm or periplasm, anchored to inner or outer membranes, or secreted into the extracellular space or even a host cell. Exposed proteins, that are on the cell surface or are secreted, are of particular biomedical interest for their potential use as novel therapeutic targets in drug development, as vaccine components, or as diagnostic markers in public health or environmental surveillance. Exposed proteins in non-pathogenic bacteria and archaea have valuable applications in agriculture and industrial processes (1,2).

Conventional laboratory approaches for high-throughput protein SCL determination can provide valuable protein localization information but are usually laborious and costly. Numerous computational or *in silico* SCL prediction methods using sequence data have been developed to complement laboratory approaches – enabling rapid localization predictions for proteins deduced from newly sequenced genomes (3). Computational protein SCL prediction methods are generally based on amino acid composition, known target sequences/motifs and sequence similarity, or a combination of the aforementioned features (4). Among the existing protein SCL predictors for bacteria and archaea (formerly collectively referred to as prokaryotes), PSORTb, is one of the most widely used SCL predictors, and has remained the most precise bacterial SCL predictor since it was first made available in 2003 (5–7). Since the PSORTb 3.0 update, this predictor is capable of predicting SCL sites not only from conventional Gram-positive and Gram-negative organisms, but also from organisms with atypical cell envelope structures. Genome-wide SCL identification by PSORTb has also been shown to exceed the accuracy of high-throughput laboratory methods, such as two-dimensional gel electrophoresis and mass spectrometry (3). Such methods are prone to cross contamination of different subcellular fractions during the cell fractionation process.

**Figure 1.**
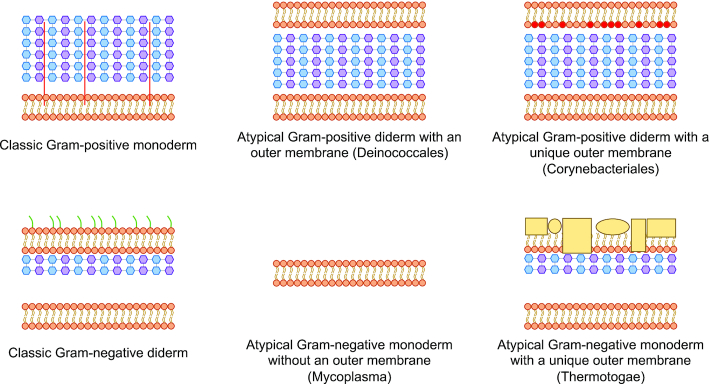
Schematic examples of types of bacterial cell envelope structures. Top left: Classic Gram-positive monoderm (containing a single cell membrane in their cell envelope, surrounded by a thick peptidoglycan layer containing lipopolysaccharides). Bottom left: Classic Gram-negative diderm (containing two cell membranes with a thin layer of peptidoglycan and a periplasmic space between the inner and outer membrane). Top middle: Atypical Gram-positive diderm with a thick peptidoglycan layer that lacks lipopolysaccharides (e.g. Deinococcales). Top right: Atypical Gram-positive diderm with a unique outer membrane (e.g. Corynebacteriales that have a mycolic acid-containing outer membrane). Bottom middle: Atypical Gram-negative monoderm without a peptidoglycan layer (e.g. Mycoplasma). Bottom right: Atypical Gram-negative diderm with a unique outer membrane (e.g. Thermotogae which contains a protein-rich outer membrane called toga). Blue hexagons representing N-acetylglucosamine and purple hexagons representing *N*-acetylmuramic acid constitute the peptidoglycan-containing cell wall. Green line represents lipopolysaccharides and red line represents lipoteichoic acid. Yellow rectangles and spheres represent structural proteins.

Many SCL predictors have been developed for proteins from organisms with the two common cell envelope structures, Gram-positive monoderms (single cell membrane) and Gram-negative diderms (double cell membrane). A classic bacterial Gram-positive monoderm comprises a cytoplasm, cytoplasmic membrane, and an external cell wall made of a thick layer of peptidoglycan. Many of the most well-studied Archaea contain these same basic components. Classic Gram-negative diderm bacteria comprise the cytoplasm, cytoplasmic membrane, a thin layer of peptidoglycan and an outer membrane. However, in some organisms, the cell envelope structures do not conform to these traditional classifications. For example, some atypical Gram-positive bacteria such as *Deinococcus* spp. (8) stain Gram-positive due to a thick peptidoglycan layer but also have an outer membrane. Likewise, some atypical Gram-negative bacteria, such as Corynebacteriales, including the notable pathogens *Mycobacterium tuberculosis* and *Mycobacterium leprae* (9), have a completely different type of outer membrane, composed of mycolic acids that stains Gram-negative or Gram-variable, even though they contain a thick peptidoglycan. Some bacteria stain Gram-negative due to a thinner peptidoglycan, but have no outer membrane, such as Mollicutes. PSORTb 3.0 (7) has incorporated SCL prediction modules for organisms with atypical cell structure, namely Gram-positive bacteria with an outer membrane and Gram-negative bacteria with no outer membrane (Figure 1).

To centralize protein SCLs determined through laboratory efforts or predicted *in silico* from genomes/genes, PSORTdb, first released in 2005, has been continually maintained and updated to accommodate the growing amount of protein SCL data (10–12). PSORTdb essentially comprises two databases: (i) ePSORTdb, containing experimentally determined protein SCLs (verified by laboratory experimentation and regularly curated from peer-reviewed literature) and (ii) cPSORTdb, containing computationally predicted SCLs using PSORTb. Both ePSORTdb and cPSORTdb are regularly updated as novel experimentally verified SCLs are curated from literature and new genomes/deduced proteomes are released from the National Centre for Biotechnology Information (NCBI) RefSeq database and incorporated into MicrobeDB (13,14).

We now report PSORTdb 4.0 (http://db.psort.org/) an expanded database featuring additional SCLs and a refresh of the web interface. A new protein sequence identification system has also been implemented in PSORTdb to address the retirement of NCBI’s GenInfo Identifier (GI) as a primary identifier. PSORTdb 4.0 builds upon PSORTdb 3.0 (still maintained at http://db3.psort.org/ for reference) by including additional manually curated protein localizations in ePSORTdb, updated protein SCL predictions in cPSORTdb, and expanded secondary localization sites. In particular, bacterial outer membrane vesicles (OMVs) have been added as a novel localization site in PSORTdb 4.0, due to the growing interest in these structures which can play a crucial role in bacteria–bacteria, and bacteria–host interactions (15).

### Enhancement of user experience for web interface for improved queries and data display

The PSORTdb website has been refreshed to feature a smoother and more modernized user interface. This new version is now hosted in Amazon Web Services infrastructure and features a range of general website updates, including the integration of Google Charts API for pie chart visualizations of protein localization breakdowns by genome. In addition, both BLAST search and other database search functionalities have been updated. The protein-specific search now facilitates quick toggling between searches in ePSORTdb and cPSORTdb datasets, and users can more easily select multiple localizations to search with. New SCLs are now included as selectable options on the PSORTdb search page.

### Implementation of novel unique protein sequence identifiers to replace retired NCBI GI numbers

The NCBI Reference Sequence (RefSeq) Database is the source of all genomes used for cPSORTdb's computationally predicted protein SCL data. Previous versions of PSORTdb used the GenInfo Identifier (GI) number system implemented by GenBank in 1994 for unique protein sequence identification. The GI number consists of a series of digits that are assigned consecutively to protein sequences as new genome sequences are deposited into NCBI (16). In 2016, NCBI phased out GIs and no longer assigns GI numbers to new protein entries. Meanwhile, in 2013, the NCBI RefSeq Database adopted a new type of protein record in which a non-redundant protein identifier is assigned to identical protein sequences found in multiple genomes (14). This novel system of identifiers follows the format of the non-redundant protein accession (‘WP_’ followed by 9 digits), a dot (‘.’), and a version number. For example, the non-redundant protein identifier of exoenzyme S from *P. aeruginosa* is ‘WP_003113791.1’. However, to identify a particular protein in a particular genome, we built upon this system. PSORTdb 4.0 has now replaced GI numbers with a combination of the RefSeq non-redundant protein accession (specifying the protein sequence), RefSeq genome/replicon accession (specifying the genome) and genome coordinates of the corresponding gene for the precise identification of a protein on a specific RefSeq genome.

### Incorporation of a new SCL concept—illustrated by outer membrane vesicles

**Figure 2.**
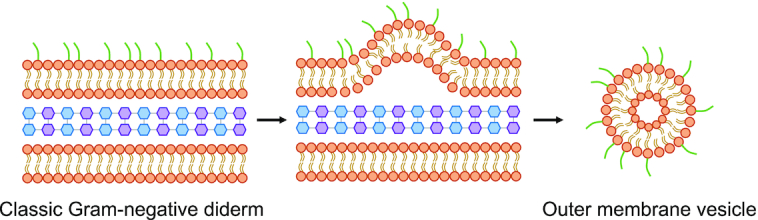
Schematic of the formation and structure of an outer membrane vesicle from the outer membrane of Gram-negative bacteria.

Since the last major published PSORTdb update (version 3.0), more SCL sites have been introduced, in addition to the primary localization sites: cytoplasm, cytoplasmic membrane, periplasm, cell wall, outer membrane, extracellular. Any expanded SCL sites (those that are not part of the primary localization sites) are considered as secondary localizations to provide a finer resolution of SCL predictions. Such secondary classification ensures primary SCL analyses are stable across database versions and compatible with other SCL databases. The most notable protein localization site newly introduced in PSORTdb 4.0 is outer membrane vesicle (OMV). OMVs are spherical buds, 20–200 nm in diameter, derived from outer membranes of bacterial diderms and mainly filled with periplasmic contents (Figure 2) (17). OMVs can contain both DNA and RNA, lipopolysaccharides, outer membrane lipids and virulence factors (15). They have evident involvement in intercellular bacterial communication, transfer of antimicrobial resistance, survival of the bacterium in host cells, and in virulence (18). The addition of OMV to the existing SCL sites is a novel concept as proteins associated with OMVs may—or may not—have their primary SCL known. Thus, the database now has the capacity to handle this possibility. The addition of such OMV SCL knowledge is important, even if the primary SCL is not known, due to increasing appreciation of the key roles OMVs can play, including in intracellular communication, and in modulating the host immune response (15).

### Expanded ePSORTdb with experimentally determined SCLs, including outer membrane vesicles

The curated ePSORTdb database has been expanded with additional 622 bacterial proteins with experimentally determined localizations, including bacterial OMVs. These novel entries, primarily from *Campylobacter jejuni* and *Pseudomonas aeruginosa* PAO1, were verified by robust laboratory experiments as reported in primary peer-reviewed literature. Proteins identified in some experiments that have less reliable methodology (resulting in potential notable cross-contamination between localizations and therefore falsely identified localizations), were not included. Among the 622 newly added ePSORTdb proteins, 293 have the OMV secondary localization. OMV proteins which do not have an experimentally determined primary localization site (cytoplasm, cytoplasmic membrane, periplasm, cell wall, outer membrane or extracellular) were assigned a primary localization of ‘Curated for secondary localization only’ to handle this new possibility. In total, ePSORTdb now encompasses over 11 000 microbial proteins with experimentally verified localizations, with the total number of proteins curated for each SCL summarized in Table 1. Regular updates of ePSORTdb are crucial for the continuous refinement of PSORTb, the SCL predictor used to compute protein SCL predictions deduced from new genomes that are regularly deposited into cPSORTdb. Likewise, ePSORTdb is a valuable resource that can aid the development and evaluation of other SCL predictors.

**Table 1.** Total number of proteins for each experimentally verified SCL site currently in the ePSORTdb dataset, grouped by type of microbe

SCL site	Gram-positive	Gram-negative	Archaea	Advanced negative^a^	Advanced positive^b^
Cytoplasmic	1619	4778	670	107	133
Cytoplasmic membrane	366	1507	85	14	19
Cell wall	77	-	18	-	8
Extracellular	303	449	27	-	10
Outer membrane	3	628	-	-	5
Periplasmic	-	543	-	-	-
Curated for secondary localization only*	-	275	-	-	-

^a^Gram-negative without an outer membrane.

^b^Gram-positive or Gram-variable, with an outer membrane.

*This is a new category of SCL site added to this version 4 update of PSORTdb in which proteins have an experimentally determined secondary localization (eg: outer membrane vesicles; OMVs), but not an experimentally confirmed primary localization. Note: Primary localizations are kept consistent (rather than adding OMVs, for example as a primary localization) to facilitate comparison between database versions, and comparative analysis or use with other resources.

### Expanded cPSORTdb for computationally predicted SCLs, using a more comprehensive predictor of cell morphology and analyzing both complete and draft bacterial and archaeal genomes

Since PSORTdb 2.0, a cell envelope classifier has been introduced to enable the automated update of cPSORTdb. This classifier predicts the appropriate PSORTb Gram-stain setting (‘Gram-positive’, ‘Gram-negative’, ‘Gram-negative without outer membrane’ or ‘Gram-positive with outer membrane’) a bacterial or archaeal deduced proteome should be analysed with, prior to SCL prediction. This classifier is based on the NCBI taxonomy, coupled with an analysis detecting several membrane markers. In brief, it first detects the presence of a classic outer membrane by detecting homologs of Omp85, the only essential outer membrane protein in Gram-negative bacteria which is critical for outer membrane function (19). Now multiple Omp85 proteins are used, reflecting diverse phyla to improve detection of homologs. If Omp85 is absent, a Corynebacteriales cutinase detector is then run to identify atypical Gram-positive bacteria with an outer membrane, or more specifically bacteria containing a Corynebacteriales mycolic acid-containing outer membrane. Predictions are then compared to the NCBI Taxonomy Database which we curated with cell structure classifications. If the results from these methods agree, the cell envelope type is automatically assigned to the genome; otherwise, manual curation is used to assign the cell envelope type. Once the bacterial cell envelope category is determined, deduced bacterial and archaeal proteomes are run through the PSORTb SCL prediction pipeline. Updated protein SCL predictions are subsequently deposited into the expanding cPSORTdb database and can be updated regularly using this cell envelope classifier. This makes cPSORTdb a valuable resource for both prediction of protein SCL as well as identification of an organism's cell envelope.

cPSORTdb now also contains the deduced proteomes from both complete and draft RefSeq genomes for the first time, considerably expanding the amount of data available (from 13 million to 66 million proteins analysed; Table 2). There are currently SCL predictions for deduced proteomes from 327 Archaea complete genomes, 38 Archaea draft assemblies, 10258 Gram-negative bacterial complete genomes, 751 Gram-negative bacteria draft assemblies, 4316 Gram-positive bacteria complete genomes, 516 Gram-positive bacteria draft assemblies, 704 Gram-positive with outer membrane complete genomes, 100 Gram-positive with outer membrane draft assemblies, 321 Gram-negative without outer membrane complete genomes and 63 Gram-negative bacteria without outer membrane draft assemblies. All protein SCL data can be queried by protein accession, protein name, organism name and several taxonomic ranks. They can also be assessed through browsing the precomputed genomes/deduced proteomes.

**Table 2.** Total number of proteins for each computationally predicted SCL site currently in the cPSORTdb dataset, grouped by type of microbe

SCL site	Gram-positive	Gram-negative	Archaea	Advanced negative^a^	Advanced positive^b^
Cytoplasmic	7927959	19650473	1448312	129664	1397908
Cytoplasmic membrane	4332420	9908830	446417	72496	643418
Cell wall	155865	-	6244	-	165
Extracellular	273479	502577	25417	4683	38755
Outer membrane	-	927950	-	-	8315
Periplasmic	-	1337237	-	-	50893
Unknown	3036149	12413292	300311	100698	1020541

^a^Gram-negative without an outer membrane.

^b^Gram-positive or Gram-variable, with an outer membrane.

## DISCUSSION

PSORTdb 4.0 represents a major update of PSORTdb, since the published update of PSORTdb 3.0 in 2016. With its greatly expanded SCL and cell structure data/analyses for bacterial and archaeal genomes, this new version includes a more comprehensive range of localization sites, with the notable addition of outer membrane vesicles that are of increasing biomedical interest for their major roles in microbial communication and host pathogenesis. However, as we learn more about the diversity of bacteria and archaea, it is clear that further study of some atypical microbial cell envelopes is needed – particularly for select phyla of the Archaea domain which are currently analysed as a whole.

The addition of analyses of deduced proteomes from draft genomes into cPSORTdb has now enabled some strains to be analysed that weren’t previously available. However, caution must be exercised in any analysis of a draft genome, as incomplete genes/deduced proteins can result in SCL mispredictions due to missing subcellular signal peptides and other SCL-related features. Draft genomes in the form of metagenome-associated genomes (MAGs) are also becoming more common. PSORTm was recently developed for prediction of SCL from metagenomics datasets (20); however, accurate long read sequences are needed to construct more complete MAGs (21) of suitable quality for inclusion into PSORTdb.

An improved PSORTdb website now provides a more user-friendly interface for navigating the updated PSORTdb database. As more microbial genomes become available, PSORTdb will continue to expand with increasing microbial protein SCL data and more refined localization sites, providing valuable resources not only for genome annotation and understanding a protein's function, but also for drug and vaccine target research. In addition, microbes in the environment are seen as attractive biomarkers of animal, plant and/or ecosystem health. There is increasing interest in monitoring fermented food/drink production, or wastewater treatment, for the presence of key microbes involved in such processes. Identifying cell surface or secreted protein markers under such conditions, as components of potential rapid, ELISA-based diagnostics, will benefit from our expanded SCL predictions, factoring in the diversity of cell envelope structures, with some that are yet to be characterized.

## DATA AVAILABILITY

PSORTdb is freely available at https://db.psort.org/ and all code and data is available under the MIT and Creative Commons licences.
